# Methanogenic Pathway and Fraction of CH_4_ Oxidized in Paddy Fields: Seasonal Variation and Effect of Water Management in Winter Fallow Season

**DOI:** 10.1371/journal.pone.0073982

**Published:** 2013-09-12

**Authors:** Guangbin Zhang, Gang Liu, Yi Zhang, Jing Ma, Hua Xu, Kazuyuki Yagi

**Affiliations:** 1 State Key Laboratory of Soil and Sustainable Agriculture, Institute of Soil Science, Chinese Academy of Sciences, Nanjing, Jiangsu, China; 2 University of Chinese Academy of Sciences, Beijing, China; 3 National Institute for Agro-Environmental Sciences, 3-1-3 Kannondai, Tsukuba, Ibaraki, Japan; University of California, Merced, United States of America

## Abstract

A 2-year field and incubation experiment was conducted to investigate δ^13^C during the processes of CH_4_ emission from the fields subjected to two water managements (flooding and drainage) in the winter fallow season, and further to estimate relative contribution of acetate to total methanogenesis (*F_ac_*) and fraction of CH_4_ oxidized (*F_ox_*) based on the isotopic data. Compared with flooding, drainage generally caused CH_4_, either anaerobically or aerobically produced, depleted in ^13^C. There was no obvious difference between the two in transport fractionation factor (*ε_transport_*) and δ^13^C-value of emitted CH_4_. CH_4_ emission was negatively related to its δ^13^C-value in seasonal variation (*P*<0.01). Acetate-dependent methanogenesis in soil was dominant (60–70%) in the late season, while drainage decreased *F_ac_*-value by 5–10%. On roots however, CH_4_ was mostly produced through H_2_/CO_2_ reduction (60–100%) over the season. CH_4_ oxidation mainly occurred in the first half of the season and roughly 10–90% of the CH_4_ was oxidized in the rhizosphere. Drainage increased *F_ox_*-value by 5–15%, which is possibly attributed to a significant decrease in production while no simultaneous decrease in oxidation. Around 30–70% of the CH_4_ was oxidized at the soil-water interface when CH_4_ in pore water was released into floodwater, although the amount of CH_4_ oxidized therein might be negligible relative to that in the rhizosphere. CH_4_ oxidation was also more important in the first half of the season in lab conditions and about 5–50% of the CH_4_ was oxidized in soil while almost 100% on roots. Drainage decreased *F_ox_*-value on roots by 15% as their CH_4_ oxidation potential was highly reduced. The findings suggest that water management in the winter fallow season substantially affects *F_ac_* in the soil and *F_ox_* in the rhizosphere and roots rather than *F_ac_* on roots and *F_ox_* at the soil-water interface.

## Introduction

Paddy fields are an important source of the greenhouse gas, methane (CH_4_), contributing to 5–19% of the total global CH_4_ emission [1]. Proper water management is considered to be one of the most important options for regulating CH_4_ emission from paddy fields [2], [3]. Generally, the fields are either intermittently irrigated or continuously flooded during the rice-growing season, and either drained without any irrigation except for rain water or kept flooded in the winter fallow season. Compared with continuous flooding, intermittent irrigation significantly decreases CH_4_ emission from rice fields during the rice-growing season by 40–70% [4]–[6]. Similarly, drainage, relative to flooding, in the winter fallow season not only prevents CH_4_ emission from the fields directly in the current season, but also sharply reduces CH_4_ emission indirectly during the following rice-growing season [7]–[9]. Although effects of water management in the winter fallow season on CH_4_ flux from the fields are considerably reported, its effect on the processes of CH_4_ emission, including CH_4_ production, oxidation and transportation, remains unclear. The stable carbon isotope technique, an important method for identifying processes of CH_4_ emission from rice fields, has been widely used through measuring carbon isotopic ratios [10]–[12]. In addition, it can be used to quantify contributions of various CH_4_ sources and provide information about carbon isotopes for global CH_4_ budget [13], [14]. To our knowledge so far, very little study has been done on the measurement of stable carbon isotopes in the fields during the rice-growing season as affected by water management in the winter fallow season.

Methanogenesis is the precondition of CH_4_ emission from paddy fields and mainly occurs through two pathways. One is H_2_/CO_2_ reduction with the participation of specific hydrogenotrophic methanogens that use H_2_ or organic molecules as H donor (CO_2_+4H_2_ → CH_4_+2H_2_O). The other is acetate fermentation with the participation of acetotrophic methanogens (CH_3_COOH → CH_4_+ CO_2_). In general, the latter plays a more important role than the former in CH_4_ formation [15], [16]. If δ^13^C-values of the CH_4_, CO_2_ and acetate involved in methanogenesis are measured, contributions of the two pathways can be estimated by using the stable carbon isotope technique [17], [18]. Theoretically, acetate fermentation and H_2_/CO_2_ reduction accounts for 67% and 33%, respectively, of the total methanogenesis. Practically, relative contributions of the two pathways vary with rice cultivar, rice growth, water management, and environmental conditions, etc. [4], [10], [11], [19]. During the rice-growing season, drainage can significantly enhance soil Eh, causing increase in oxidizing substances like Fe^3+^, sulphate and nitrate, and their inhibition of acetotrophic methanogens, thus reducing acetate-dependent methanogenesis [4], [20]. In the winter fallow season, water management also significantly affects soil Eh, CH_4_ production and then CH_4_ emission from the fields during the following rice-growing season [8], but its impact on relative contributions of the two main pathways of methanogenesis remains poorly known.

CH_4_ oxidation, which occurs at the root–soil interface and soil–water interface, is very important to regulating paddy CH_4_ emission. By comparing CH_4_ emission from the field or CH_4_ production from aerobic incubation with methanogenesis in the strict anaerobic environment at the early stage, it was found that as much as 50–90% of the CH_4_ was oxidized before escaping into the atmosphere [21]–[23]. By using the stable carbon isotope method to quantify the fraction of CH_4_ oxidized in the paddy fields, recent studies in America and Italy indicated that it was less than 50% [10], [12], [24], [25]. In China however, the fraction of CH_4_ that was oxidized in a paddy field under intermittent irrigation during the rice-growing season was measured by this means to be up to 80% [4]. It was significantly higher than those in the fields under continuous flooding as above mentioned. Moreover, CH_4_ oxidation potential was relatively higher in intermittently irrigated paddy soil than in continuously flooded soil [4], which suggests that CH_4_ oxidation is highly impacted by water management during the rice-growing season. It is further indicated that oxidization of endogenous CH_4_ in the paddy fields seems to be more obvious in China, particularly in the fields that are intermittently irrigated during the rice-growing season. Although CH_4_ oxidation potential in paddy soil in a whole year has been reported [9], the percentage of CH_4_ oxidized in the field as affected by water management in the winter fallow season is still unknown.

Therefore, a 2-year field and incubation experiment was carried out in the paddy fields subjected to two types of water management (flooding and drainage) in the winter fallow season. Seasonal CH_4_ emission fluxes, CH_4_ in soil pore water and floodwater, CH_4_ in the aerenchyma of the plants, CH_4_ production and oxidation in fresh paddy soil and rice roots, and their respective δ^13^CH_4_-values during the rice-growing season were measured. The objectives of this study were: (1) to investigate impact of water management in the winter fallow season on CH_4_ production, oxidation and emission and their δ^13^CH_4_; and (2) further to evaluate its effect on pathways of CH_4_ production and fraction of CH_4_ oxidized in the fields by using the isotopic measurements.

## Materials and Methods

### Field Description and Experimental Design

With the authorization of the Institute of Agricultural Science, Zhenjiang City, the experiment was carried out at Baitu Town, Jurong City, Jiangsu Province, China (31°58′N, 119°18′E). Main features of the experiment field have already been described in detail before [8]. The experiment was designed to have two treatments, three replicates each, i.e. winter fallow under continuous flooding (Flooding) and winter fallow without irrigation except for rain water (Drainage). Measurements of this study were performed during the 2008 and 2009 rice-growing seasons. Rice stubble and wild weeds were all removed from the experimental plots after rice harvest in the winter fallow season. For rice transplanting in the next rice-growing season, all the plots were ploughed the way the local farmers do. During the rice-growing season, they were continuously flooded and only drained for rice harvest. Rice seedlings (Cultivar “Oryza sativa L. Huajing 3”) were transplanted at their 3–4-leaf stage on June 22 and 26 in 2008 and 2009, respectively. Urea was applied at a rate of 300 kg N ha^−1^, of which 50% was done as basal fertilizer, 25% as tillering fertilizer, and 25% as panicle fertilizer. Both Calcium superphosphate and Potassium chloride were applied as basal fertilizer at a rate of 450 and 225 kg ha^−1^, respectively. For further details of the farming practices during the two years, please see Zhang et al. [8].

### Field Sampling and Measuring

CH_4_ flux was observed using the static closed chamber method [8]. To measure the flux, gas samples were collected at 4–7-day intervals using 18 mL vacuum vials. For determining isotopic signature (δ^13^C) of the emitted CH_4_, gas samples were taken at 10∼20–day intervals using 500 mL bags (Aluminium foil compound membrane, Delin gas packing Co., Ltd, Dalian, China) with a small battery-driven pump [26]. The first sample was collected after the chamber was closed for 3–5 min, and the second at the end of the 2 h closure. Isotopic signature (*S*) of the emitted CH_4_ was calculated using the equation below:
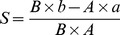
(1)where *A* and *B* stands for CH_4_ concentration (µl L^−1^) in the samples at the beginning and at the end, respectively, while *a* and *b* for the corresponding δ^13^CH_4_-values (‰) of the gas samples, separately. Simultaneously, soil redox potential (Eh) at the depth of 10 cm was measured, using Pt-tipped electrodes (Hirose Rika Co. Ltd., Japan) and an oxidation-reduction potential meter with a reference electrode (Toa PRN-41). Soil temperature at the depth of 10 cm was measured with a hand-carried digital thermometer (Yokogawa Meter and Instruments Corporation, Japan).

Soil pore water samples were collected using a Rhizon soil moisture sampler (10 RHIZON SMSMOM, Eijkelkamp Agrisearch Equipment, Giesbeek, Netherlands) [26]. The samplers were installed (in triplicate) in the plots prior to rice transplanting and then left in the soil over the whole observational periods. Samples (∼5 mL) were firstly extracted using 18 mL vacuum vials to flush and purge the sampler before sampling. Then ∼10 mL of soil solution was drawn into another vial for further analysis. Simultaneously, 10 mL of floodwater was collected using a plastic syringe and then transferred in to an 18 mL vacuum vial. Finally, the pressure of all sampling vials was equilibrated by filling in pure N_2_ gas. After heavy shaking by hand, the airs in the headspace of the vials were directly analyzed for CH_4_ on the GC-FID, and their corresponding δ^13^CH_4_-values were determined using the isotope ratio mass spectrometer. CH_4_ concentrations (*C_CH_*_4_) in pore water and floodwater were calculated using the following equation:
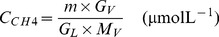
(2)where *m* stands for mixing ratio of CH_4_ in the headspace of a vial (µL L^−1^), *M_V_* for volume of an ideal gas (24.78 L mol^−1^ at 25°C), *G_V_* for volume of the gas headspace of the vial (L), and *G_L_* for volume of the liquid in the vial (L).

Samples of the gas in the aerenchyma of and emitted from the plants were taken using specially designed PVC bottomless pots [27]. The pot, 30 cm in height and 17 cm in diameter, was designed to have a water-filled trough around its top, avoiding any possible gas exchange during the sampling times. A PVC plate (18 cm in diameter) with a hole (the diameter could be adjusted to the growing plant) in the center was placed on top of each pot, allowing the plant to grow through the hole and keep divided into two parts. Then, one plant inside the pot was cut right above the plate while the other remained intact as the control. Finally, chambers (30×30×100 cm) were laid on the pots, and gas samples in the headspace of the chambers were collected simultaneously with a small battery-driven pump.

Triplicate soil cores were collected from each experimental plot using a stainless steel corer (7 cm diameter×25 cm length) and then prepared into mixture [9]. Samples (in triplicate) from the mixture, about 50 g (dry weight) each, were taken and promptly transferred into 250-mL Erlenmeyer flasks separately, and turned into slurries with N_2_-flushed de-ionized sterile water at the ratio of 1∶1 (soil/water). During the whole process, the samples were constantly flushed with N_2_ to remove O_2_ and CH_4_, and the flasks containing these samples were sealed for anaerobic incubation. Some other flasks with air headspace were sealed directly for aerobic incubation. They were all stored in N_2_ at 4°C for further analysis within 8 h. A small portion of the soil sample was dried for 72 h at 60°C for determination of isotopic composition of the organic carbon.

Complete rice plants together with roots were carefully collected from the experimental plots at each main rice growth stage, i.e. tillering (TS), booting (BS), grain-filling (FS) and ripening (RS) stages, in 2009 [8]. The roots were washed clean with N_2_-flushed demineralized water and cut off from the green shoots at 1–2 cm from the root with a razor blade. The fresh roots, 20 g each portion, were then put into flasks, separately, for further preparation and processing in the same way as for the soil. The shoots were dried at 60°C for 72 h for dry weight measurement and then stored at room temperature for determination of isotopic composition of the organic carbon.

### Fresh Soil and Roots Incubations

CH_4_ production potentials were measured for the paddy soil and rice roots under anaerobic incubation. The flasks were flushed with N_2_ consecutively for six times through double-ended needles connecting a vacuum pump to purge the air in the flasks of residual CH_4_ and O_2_. Simultaneously, methanogenesis was determined aerobically using flasks with air headspace directly. They were subsequently incubated at a temperature the same as measured in the field for 50 h in darkness. Gas samples were collected twice with a pressure lock syringe, and analyzed 1 h and 50 h later after the flasks were heavily shaken by hand, for CH_4_ on the GC-FID. CH_4_ production was calculated using the linear regression of CH_4_ increasing with the incubation time.

CH_4_ oxidation potentials were determined for the paddy soil and rice roots under aerobic incubation with high CH_4_ concentration supplemented, using equipment the same as described above. Firstly, pure CH_4_ was injected into each flask to make a high concentration inside (∼10000 µL L^−1^). Then, the flasks were incubated in dark under the same temperature as measured in the field and shaken at 120 r.p.m. CH_4_ depletion was measured by sampling the headspace gas in the flask after vigorous shaking for subsequent GC-FID analysis. The first sample was collected generally 30 min after pure CH_4_ was injected. Samples were then taken in 2–3 h intervals during the first 8 h of the experiment. They were left in the flasks over night and measured the next day in 2 h intervals again. CH_4_ oxidation was calculated by linear regression of CH_4_ depletion with incubation time.

### Analytical Methods

CH_4_ was quantified using the gas chromatograph (GC) equipped with a flame ionization detector (FID) [28]. The isotopic composition (δ^13^C) of CH_4_ and CO_2_ was determined with a Finnigan MAT-253 Isotope Ratio Mass Spectrometer (IRMS, Thermo-Finnigan, Bremen, Germany) using the continuous flow technique [26], [29]. The IRMS had a fully automatic interface for pre-GC concentration (Pre-Con) of trace gases, and the precision of repeated analyses was ±0.196‰ (n = 9) with 2.02 µL L^−1^ CH_4_ injected. Gas samples were first blown into the chemical trap with Mg(ClO_4_)_2_ and ascarite by He flow (20 mL min^−1^). Over 99.99% of the CO_2_ and H_2_O in the samples was absorbed and removed. CH_4_ in the samples was then converted into CO_2_ in a combustion reactor at about 1000°C. Subsequently, it was flowed into the freezing traps with liquid nitrogen (–196°C) and the GC for further separation. The separated gases were finally transferred into the mass spectrometer for δ^13^C determination. The dried soil and plant samples were analyzed for carbon isotope composition with a Finnigan MAT-251 Isotope Ratio Mass Spectrometer (Thermo Finnigan, Bremen, Germany). Soil organic carbon contents were measured by wet oxidation using dichromate in acid medium followed by the FeSO_4_ titration method.

### Calculations

Isotope ratios are expressed in the standard delta notation:
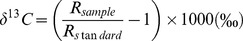
(3)where *R* stands for ^13^C/^12^C of the sample and the standard, respectively, using PDB carbonate for the standard. Carbon isotope fractionation factor during acetate fermentation (*ε_acetate/CH4_*) or H_2_/CO_2_ reduction (*α_CO2/CH4_*) for methanogenesis was defined by Hayes [30]:



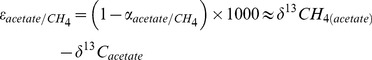
(4)

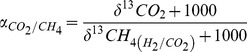
(5)where δ^13^C_acetate_, δ^13^CH_4 (acetate)_ and δ^13^CH_4 (H2/CO2)_ is the δ^13^C values of acetate, CH_4_ produced from acetate and from H_2_/CO_2_, respectively.

Relative contribution of acetate to total CH_4_ (*F_ac_*) was calculated using the following mass balance, assuming that acetate fermentation and H_2_/CO_2_ reduction were the only sources of methanogenesis in the rice fields [10]-[12]:
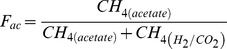
(6)

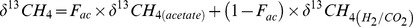
(7)where δ^13^CH_4_ stands for δ^13^C value of total CH_4_. In addition, the fraction of CH_4_ that was oxidized (*F_ox_*) in the fields was estimated using the equation given by Stevens and Engelkemeir [13] and Tyler et al. [12]:
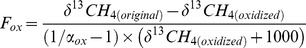
(8)where δ13CH_4(original)_ stands for carbon isotopic signature of the primarily produced CH_4_, δ^13^CH_4__(oxidized)_ for carbon isotopic signature of the residual CH4 after oxidization, of which the calculation was done using a semi-empirical equation [12]:
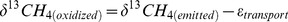
(9)and α_ox_ stands for isotope fractionation factor due to CH_4_ oxidation by the methanotrophs, and ε_transport_ for isotope fractionation factor due to CH_4_ transport by the plants.

### Statistics

Statistical analysis was done using the SPSS 18.0 software for Windows (SPSS Inc., Chicago). Differences between the two treatments in mean (n = 3) CH_4_ concentration, mean CH_4_ production and oxidation potentials, and mean soil Eh were determined through one-way analysis of variance (ANOVA) and least significant difference (LSD) test. Relationships between CH_4_ fluxes and emitted δ^13^CH_4_ (n = 18), between mean CH_4_ production potential and soil Eh (n = 11), and between CH_4_ oxidation potential and soil temperature (n = 11) were assessed using correlation analysis. Statistical significant differences and correlations were set at *P*<0.05.

## Results

### CH_4_ Emission and δ^13^C

**Figure 1:**
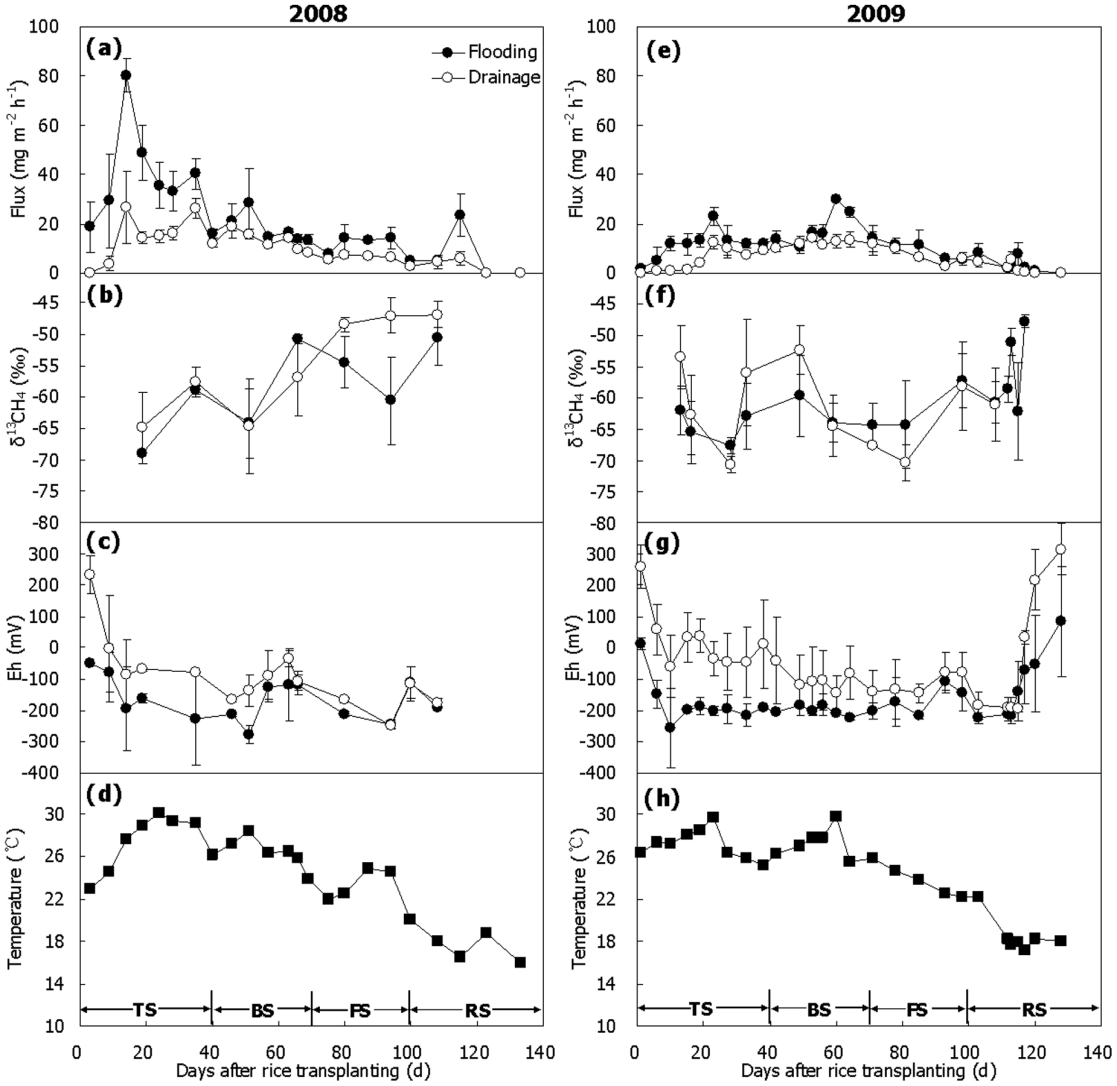
Seasonal variations of CH_4_ emission, δ^13^C-value of emitted CH_4_, soil Eh and soil temperature. (a, b, c and d) 2008, (e, f, g and h) 2009. TS, BS, FS and RS represent tillering, booting, grain-filling and ripening stages, respectively. Bars represent standard errors (n = 3).

CH_4_ emissions (Fig. 1a, e) were significantly higher from flooded fields than from drained fields as had been reported before [8]. Different variation patterns were observed in the δ^13^C of the emitted CH_4_ in 2008 and 2009 seasons (Fig. 1b, f). Generally, the emitted CH_4_ tended to be ^13^C-enriched in 2008 with its δ^13^C-value increased from –69 to –51‰ in Treatment Flooding, and from –65 to –47‰ in Treatment Drainage (Fig. 1b). However, more complicated changes were observed in 2009, showing that the emitted CH_4_ was relatively enriched in ^13^C at the beginning and at the end of the season, and relatively ^13^C-depleted in the middle of the season (Fig. 1f). However, little difference was found between Treatments Flooding and Drainage, with δ^13^C-values being in the range of –68 ∼ –48‰ and –71 ∼ –53‰, respectively (Fig. 1b, f, *P*>0.05). Although more measurements were performed in 2009 than in 2008, the mean δ^13^C-value seemed to be more positive in 2008 (–58 ∼ –55‰) than in 2009 (–62 ∼ –61‰). Notably, negative relationship was observed between CH_4_ flux and δ^13^C in the seasonal variation in 2008 (r = –0.695, *P*<0.01) and 2009 (r = –0.546, *P*<0.01).

Significant difference in soil Eh was observed between the two treatments (Fig. 1c, g, Table 1). Soil Eh was very close to 0 mV at the beginning of the season and remained much lower in Treatment Flooding than in Treatment Drainage throughout the two seasons, with the averaged value of –165 and –88 mV in 2008, and –153 and –26 mV in 2009, respectively. Soil temperature at the depth of 10 cm generally peaked around D25 (25 days after rice transplanting) and then gradually declined till the end of the season (Fig. 1d, h), fluctuating within the range from 16 to 30.1°C in 2008 and from 17.2 to 29.7°C in 2009, and being averaged 24.3 and 24.4°C, respectively.

**Table 1:** Mean CH_4_ concentration (µmol L^−1^) in soil pore water, mean CH_4_ production and oxidation potentials (µgCH_4_ g d^−1^), and mean soil Eh (mV) during the 2008 and 2009 rice seasons (mean ± SD, n = 3).

*Treatment*	*Concentration*	*Production*		*Oxidation*		*Eh*
		Soil	Roots	Soil	Roots	
2008						
Flooding	340±47 a	2.96±0.30 a	–	8.66±1.64 a	–	–165±15 a
Drainage	281±63 a	1.16±0.26 b	–	8.37±1.24 a	–	–88±26 b
2009						
Flooding	170±6 a	1.99±0.11 a	27.2±3.3 a	5.99±0.28 a	649±88 a	–153±16 a
Drainage	119±12 b	1.15±0.02 b	11.9±2.1 b	6.66±1.39 a	308±59 b	–26±15 b

Means in the same column followed by different letters between the two treatments indicate significant difference at *P*<0.05.

### CH_4_ Concentration and δ^13^C

**Figure 2:**
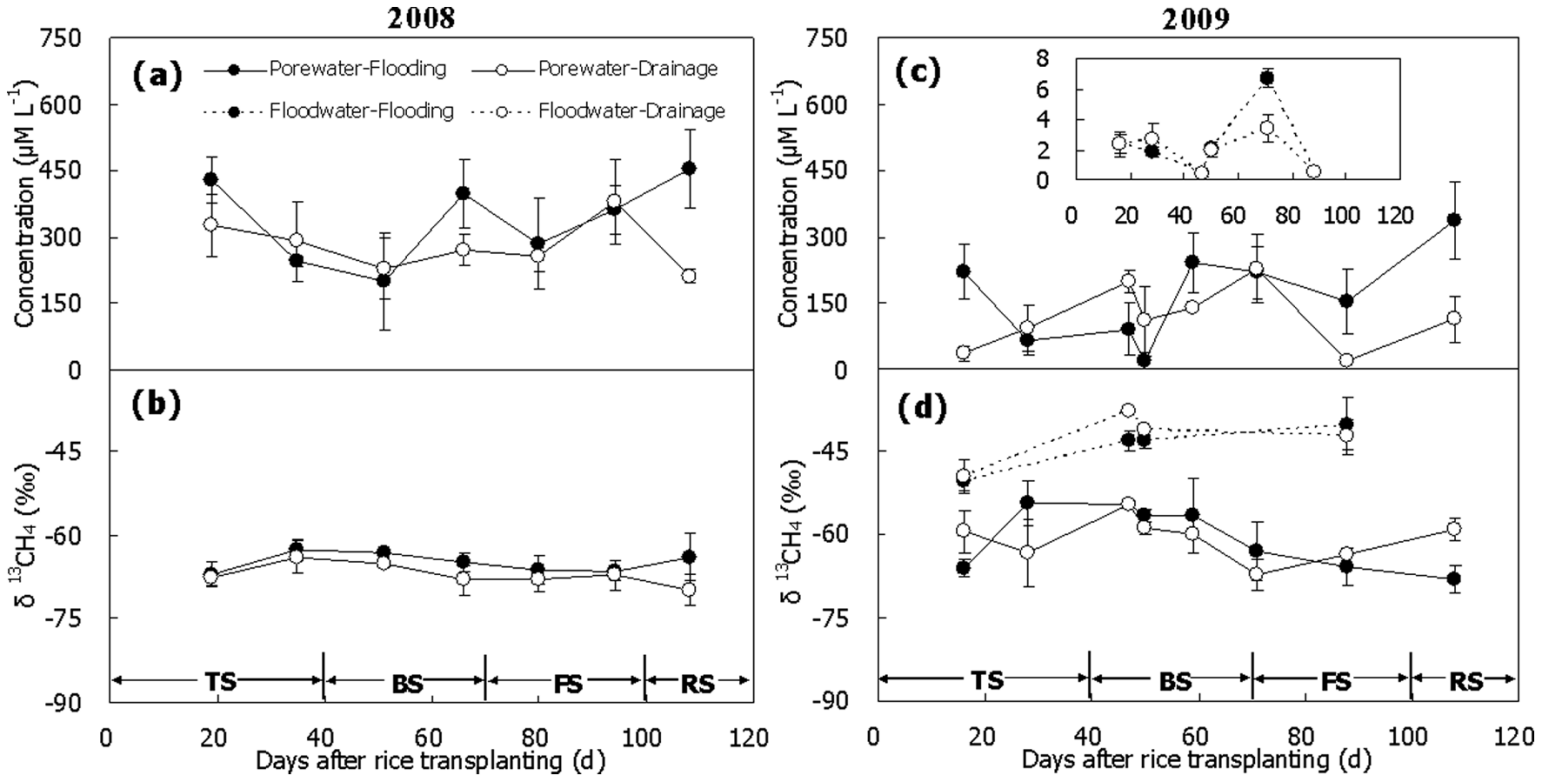
Temporal variation of concentration and δ^13^C-value of CH_4_ in soil pore water and floodwater. (a, b) 2008, (c, d) 2009. TS, BS, FS and RS represent tillering, booting, grain-filling and ripening stages, respectively. Bars represent standard errors (n = 3).

CH_4_ concentration in pore water for Treatment Flooding was relatively high (200–400 µmol L^−1^) at the beginning of the season, dropped subsequently to 20–200 µmol L^−1^ and then turned upwards again to 350–450 µmol L^−1^ at the end of the season in both 2008 and 2009 (Fig. 2a, c). For Treatment Drainage however, CH_4_ concentration decreased gradually from 300 to 200 µmol L^−1^ during the 2008 season (Fig. 2a), whereas it was the highest (∼200 µmol L^−1^) in the middle and the lowest (<50 µmol L^−1^) at the beginning and the end of the 2009 season (Fig. 2c). The averaged CH_4_ concentration during the two seasons was generally higher in Treatment Flooding than in Treatment Drainage (Table 1). δ^13^C-value of the CH_4_ was relatively stable during the 2008 season though it increased and then slightly decreased (Fig. 2b). As a whole, CH_4_ was much more ^13^C-enriched in Treatment Flooding (–65‰) than in Treatment Drainage (–67‰) over the 2008 season (Fig. 2b, *P*<0.05). In the 2009 season however, δ^13^C-value fluctuated sharply within the range from –65 to –55‰ to –70‰ or so (Fig. 2d). No obvious difference in mean δ^13^C-value (∼ –60‰) was observed between the two treatments in 2009 (Fig. 2d, *P*>0.05).

CH_4_ concentration in floodwater of the field in 2009 was measured simultaneously. No more than 7 µmol L^−1^ of CH_4_ was detected though little data were obtained (Fig. 2c). On the other hand, CH_4_ in floodwater became more and more ^13^C-enriched towards the end of the season, with the δ^13^C-value increased from –50 to –40‰ (Fig. 2d). Little difference in δ^13^C-value was observed as well between Treatments Flooding and Drainage (Fig. 2d, *P*>0.05). Compared with porewater CH_4_, floodwater CH_4_ was much more enriched in ^13^C (Fig. 2d, *P*<0.05).

### Plants Emitted and Aerenchymatic CH_4_ and δ^13^C

To quantify stable carbon isotope fractionation during the CH_4_ emitted through the aerenchyma of the plants, δ^13^C-values of the emitted CH_4_ and aerenchymatic CH_4_ were measured simultaneously. On the three sampling days during the 2009 season, the emitted CH_4_ was relatively stable with its δ^13^C-value stable around –60‰ (Table 2). The aerenchymatic CH_4_ as expected, was significantly ^13^C-enriched compared to the emitted CH_4_, with the δ^13^C-values varying in the range of –51 ∼ –42‰, and being about –47‰ on average for the two treatments (Table 2). As a result, the isotope fractionation factor due to CH_4_ transport (*ε_transport_*) was determined to be in the range from –16 to –11‰ in Treatment Flooding and from –14 to –12‰ in Treatment Drainage. As a whole, no obvious difference in mean value of *ε_transport_* (∼ –13‰) was observed between the two treatments (Table 2, *P*>0.05).

**Table 2:** δ^13^C-values of CH_4_ (‰) in the aerenchyma of and emitted from the plants during the 2009 rice season.

Days after ricetransplanting (d)	Aerenchymatic CH_4_ (a)		Emitted CH_4_ (b)		*ε_transport_* = b–a	
	Flooding	Drainage	Flooding	Drainage	Flooding	Drainage
37	–48.46	–51.49	–60.94	–63.82	–12.48	–12.33
62	–42.44	–43.58	–58.77	–57.84	–16.33	–14.27
98	–48.56	–47.32	–59.51	–59.18	–10.95	–11.86
Mean	–46.49	–47.46	–59.74	–60.28	–13.25	–12.82
SD	3.50	3.96	1.10	3.14	1.28	2.77

### CH_4_ Production Under Anaerobic Incubation and δ^13^C

**Figure 3:**
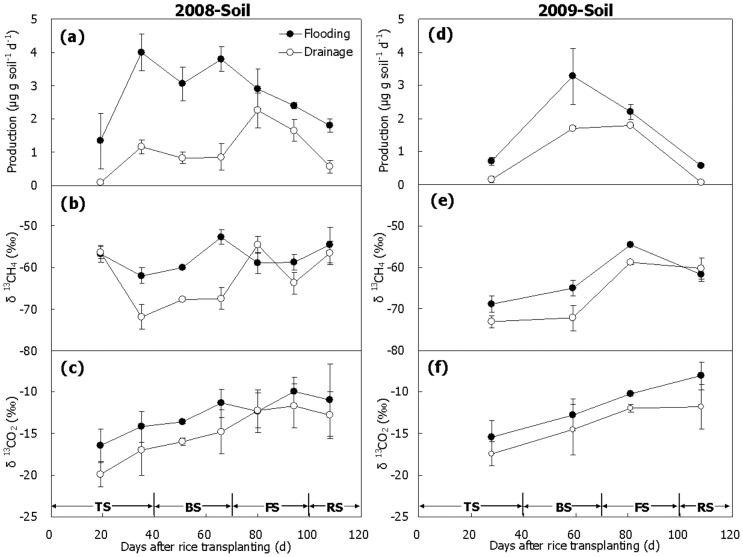
CH_4_ production potential and δ^13^C-values of CH_4_ and CO_2_ anaerobically produced in paddy soil. (a, b and c) 2008, (d, e and f) 2009. TS, BS, FS and RS represent tillering, booting, grain-filling and ripening stages, respectively. Bars represent standard errors (n = 3).

CH_4_ production potentials of the slurries of paddy soil were measured during the 2008 and 2009 rice seasons (Fig. 3a, d). Methanogenesis started more quickly and became more intense in Treatment Flooding than in Treatment Drainage over the two seasons (Fig. 3a, d), peaked around D40–60 for the former and around D80 for the latter. Mean production potential was significantly higher in Treatment Flooding than in Treatment Drainage during the two seasons (Table 1, *P*<0.05). The produced CH_4_ was relatively stable in δ^13^C (∼ –60‰) in Treatment Flooding while fluctuated sharply (from –72 to –55‰) in Treatment Drainage in 2008 (Fig. 3b). In 2009 however, it was gradually enriched in ^13^C for the two treatments, with δ^13^C-value ranging from –70 to –60‰ (Fig. 3e). In addition, the mean δ^13^CH_4_-value in Treatment Flooding appeared to be slightly more positive than that in Treatment Drainage over the two seasons, varying in the range of –63 ∼ –58‰ and –66 ∼ –63‰, respectively. The produced CO_2_ became isotopically heavier step by step, causing δ^13^C-value to decrease from –20 ∼ –15‰ at the beginning of the season to around –10‰ at the end of the season, and it was relatively more ^13^C-enriched in Treatment Flooding than in Treatment Drainage over the two seasons (Fig. 3c, f, *P*>0.05).

**Figure 4:**
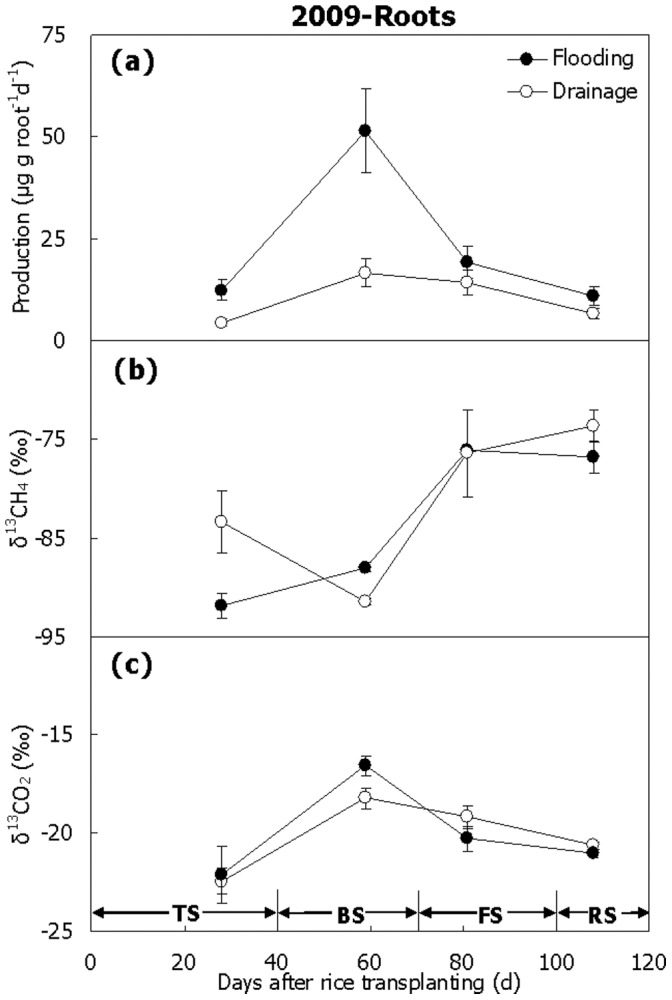
CH_4_ production potential and δ^13^C-values of CH_4_ and CO_2_ anaerobically produced on rice roots. (a) Potential, (b) δ^13^CH_4_, (c) δ^13^CO_2_. TS, BS, FS and RS represent tillering, booting, grain-filling and ripening stages, respectively. Bars represent standard errors (n = 3).

Abundant methanogenesis was measured on the fresh rice roots under anaerobic incubation in 2009 (Fig. 4a). The production of CH_4_ increased sharply and peaked around D60, just like the soil (Fig. 3d). Then it decreased gradually till the end of the season. As a whole, it was significantly higher in Treatment Flooding than in Treatment Drainage (Fig. 4a, Table 1, *P*<0.05). Similar to CH_4_ produced in the soil, CH_4_ produced on the roots was depleted in ^13^C at the beginning of the season (Fig. 4b). Subsequently, it became more ^13^C-enriched, with its δ^13^C-values ranging from –90 to –75‰. No significant difference was observed in mean δ^13^C-value between Treatment Flooding (–83‰) and Treatment Drainage (–81‰). However, it was much more negative compared to the CH_4_ produced in the soil in δ^13^C-value (Figs. 3e and 4b, *P*<0.01). The δ^13^C-value of produced CO_2_ ranged from –22 to –17‰ over the two seasons and no obvious difference was observed between the two treatments (Fig. 4c, *P*>0.05).

### CH_4_ Production Under Aerobic Incubation and δ^13^C

**Figure 5:**
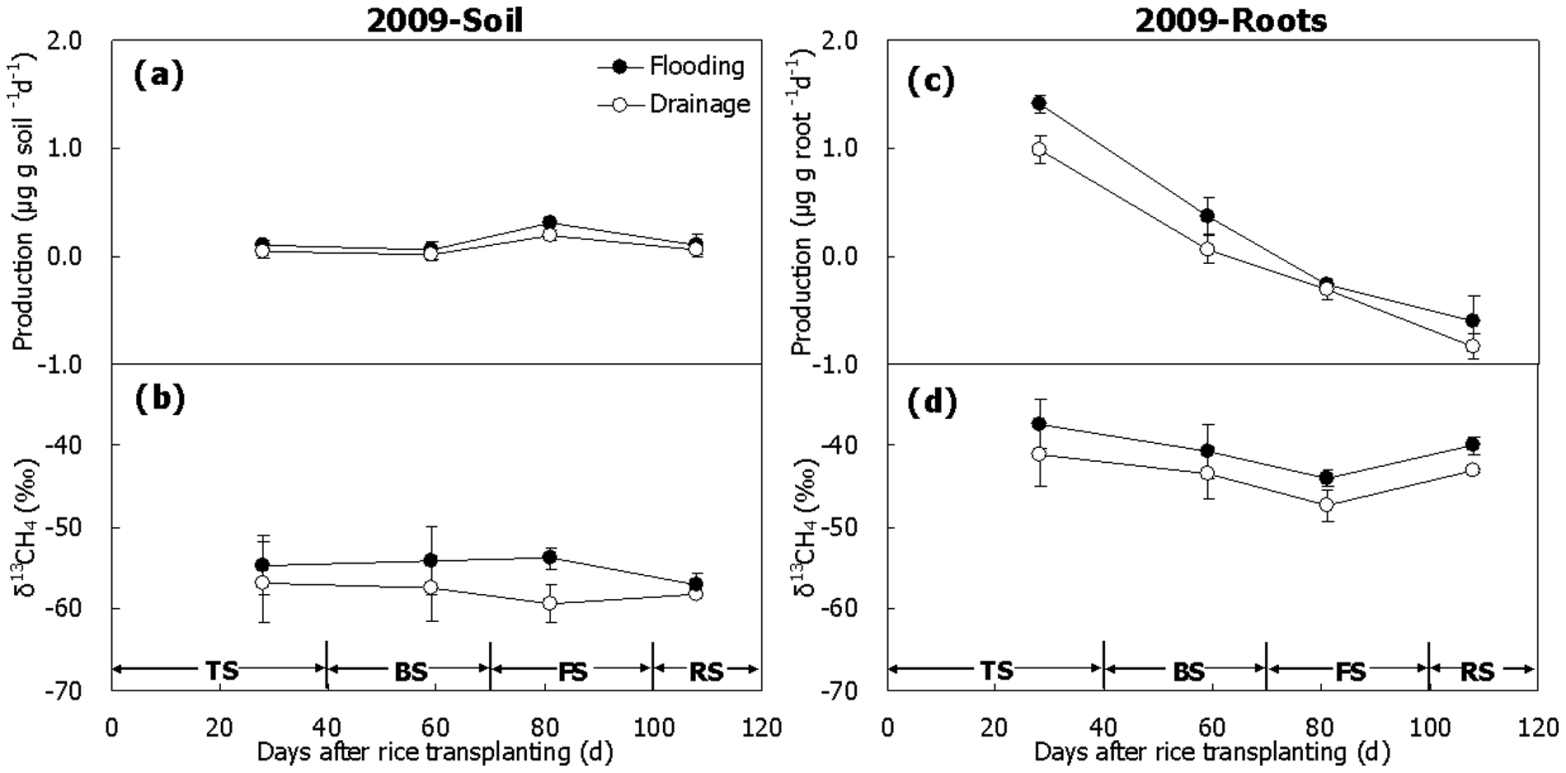
Temporal variation of CH_4_ production and corresponding δ^13^C-value in aerobic incubation. (a, b) Paddy soil, (c, d) Rice roots. TS, BS, FS and RS represent tillering, booting, grain-filling and ripening stages, respectively. Bars represent standard errors (n = 3).

Less than 0.3 µgCH_4_ gsoil^−1^ d^−1^ was produced in the soil under aerobic condition over the 2009 season (Fig. 5a), and 1.0–1.5 µgCH_4_ groots^−1^ d^−1^ was on the roots at the beginning of the season and below 0 µgCH_4_ groots^−1^ d^−1^ at the end of the season (Fig. 5c). The produced CH_4_ was very stable over the season both in the soil and on the roots, with δ^13^C-values around –58 ∼ –55‰ and –44 ∼ –41‰, respectively. Opposite to the CH_4_ produced under anaerobic condition, it was significantly more enriched in ^13^C on the roots than in the soil (Fig. 5b, d, *P*<0.01). Generally, the δ^13^C-value was more positive in Treatment Flooding than in Treatment Drainage (Fig. 5b, d). In addition, the CH_4_ produced under aerobic condition was significantly ^13^C-enriched relative to that produced under anaerobic condition, especially those from the roots (Figs. 3e, 4b and 5b, d, *P*<0.01).

### CH_4_ Oxidation Under Aerobic Incubation Amended with High CH_4_ Concentration

**Figure 6:**
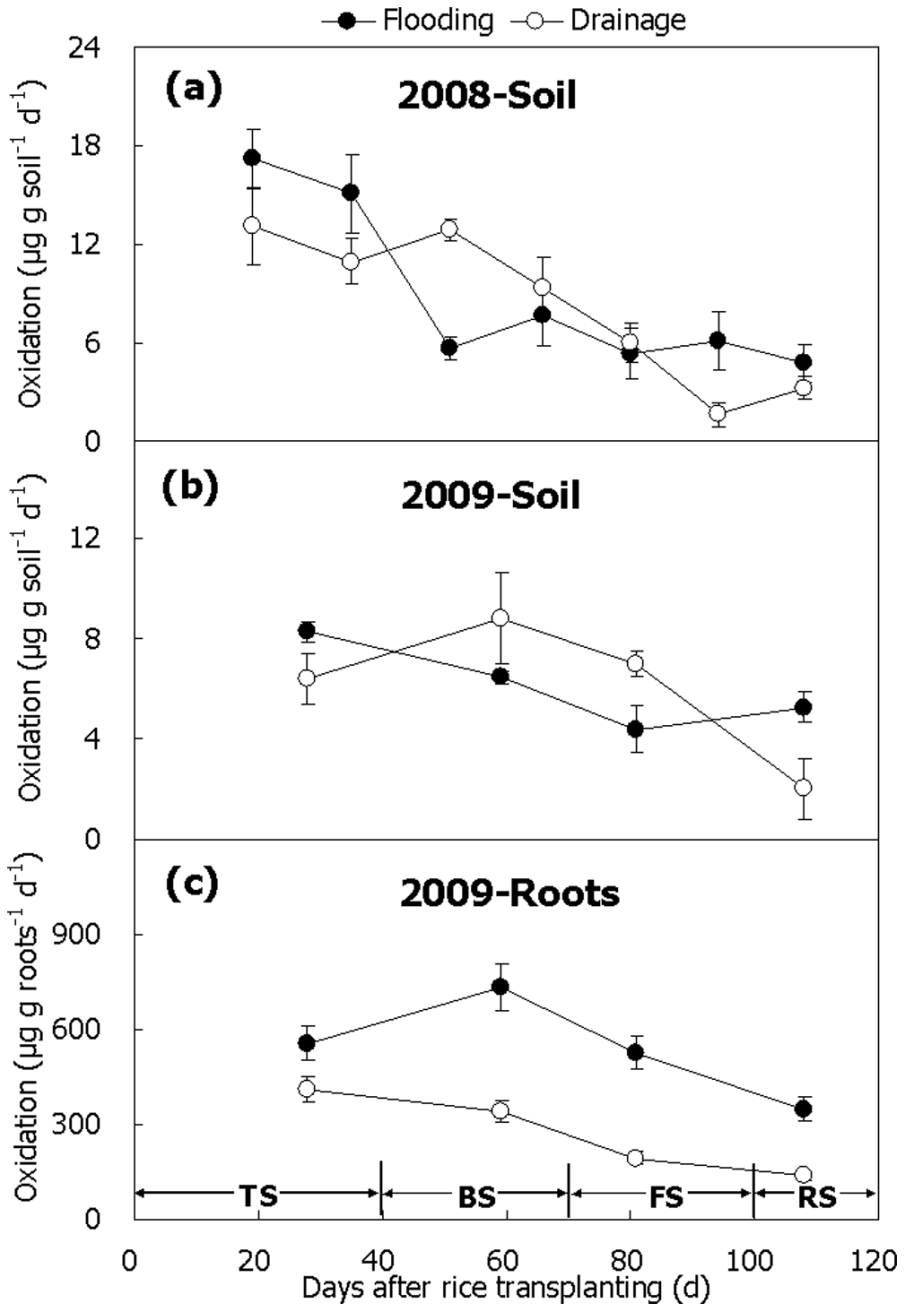
Temporal variation of CH_4_ oxidation potential in paddy soil and on rice roots. (a) 2008, (b and c) 2008 and 2009. TS, BS, FS and RS represent tillering, booting, grain-filling and ripening stages, respectively. Bars represent standard errors (n = 3).

Similar variation patterns of the CH_4_ oxidation potentials in the paddy soil during the 2008 and 2009 seasons were observed, showing a peak at the beginning of the season and a steep slope till the end of the season (Fig. 6a, b). Although the potential was relatively lower in Treatment Flooding than in Treatment Drainage in the middle of the season but slightly higher both at the beginning and at the end of the season (Fig. 6a, b), no significant difference was observed between the two treatments in mean of the potential (Table 1, *P*>0.05). Notably, a significant positive relationship was observed between CH_4_ oxidation potential and soil temperature in temporal variation (r = 0.703–0.859, *P*<0.05). Intensive oxidation signal on the fresh roots was observed, which was also the strongest (400–600 µgCH_4_ groots^−1^ d^−1^) at the beginning of the season and declined to the lowest (150–400 µgCH_4_ groots^−1^ d^−1^) at the end of the season (Fig. 6c). Throughout the 2009 season, CH_4_ oxidation potential on the roots was significantly higher in Treatment Flooding than in Treatment Drainage (Fig. 6c, *P*<0.05).

### Organic Carbon in Soil and Plant Samples

During the 2008 season, the content of organic carbon in the soil was 1.02±0.08% in Treatment Flooding and 1.11±0.05% in Treatment Drainage, and it seemed to increase during the 2009 season, reaching 1.65±0.01% and 1.83±0.10%, respectively. Soil organic carbon in Treatment Drainage was very stable in δ^13^C (–27.9‰) during the two rice seasons, whereas it was slightly ^13^C-enriched in Treatment Flooding, with δ^13^C-value increasing from –28.1‰ in 2008 to –27.0‰ in 2009. Organic carbon in the plant samples showed little change throughout the 2009 season, with δ^13^C-value of –28.9‰, –29.2‰ and –28.7‰ on D27, D66 and D108, respectively, although it was slightly lighter in contrast to the organic carbon in the soil.

## Discussion

### Effects on Stable Carbon Isotopes

**Figure 7:**
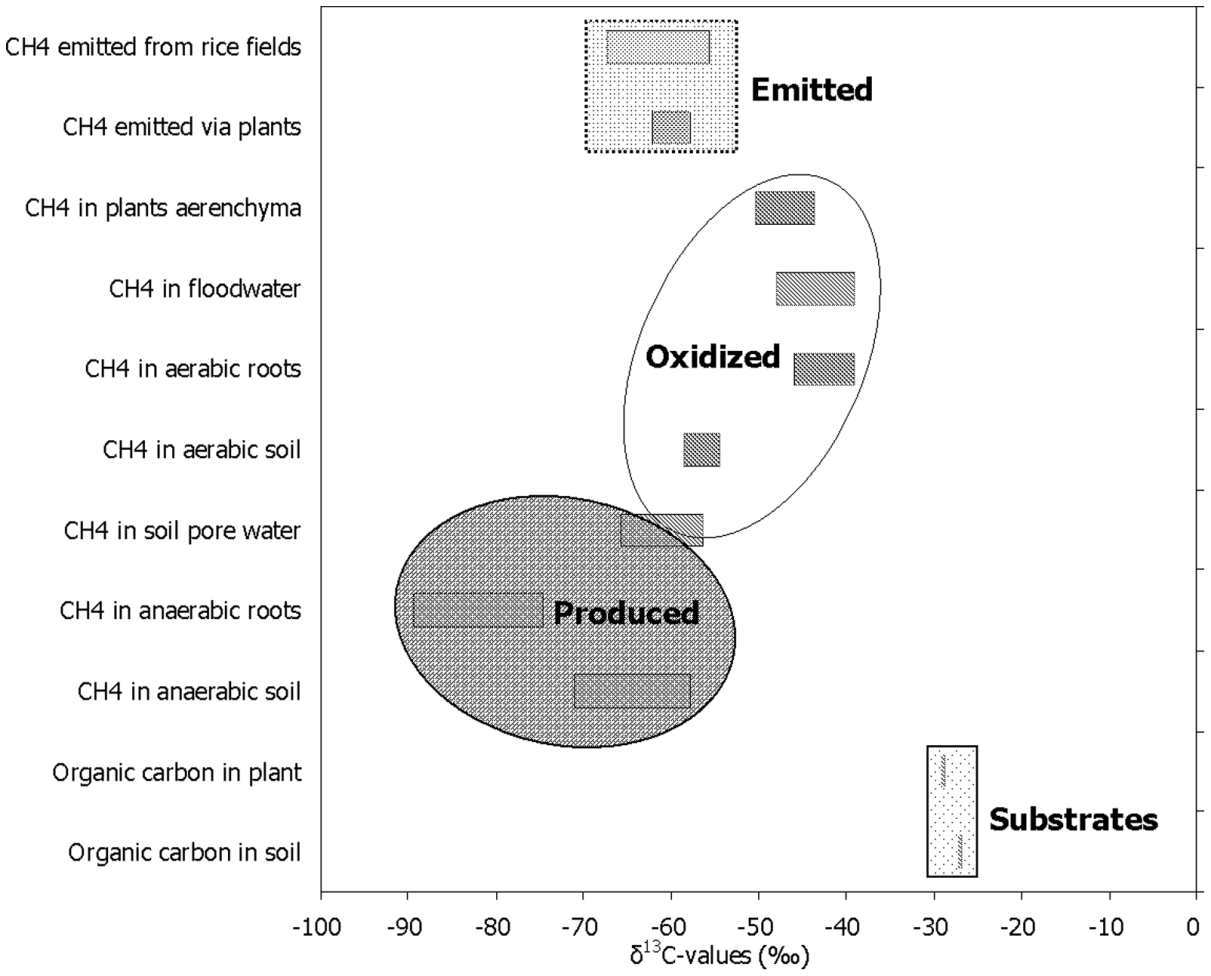
Stable carbon isotopes in organic carbon and of CH_4_ in 2009. Stable carbon isotopes in the soil and plant organic carbon and CH_4_ isotopic compositions in the processes of CH_4_ emission from the paddy fields and in the lab conditions. The δ^13^CH_4_-value of each component was given in the range of isotopic variation during the 2009 rice season.

**Figure 8:**
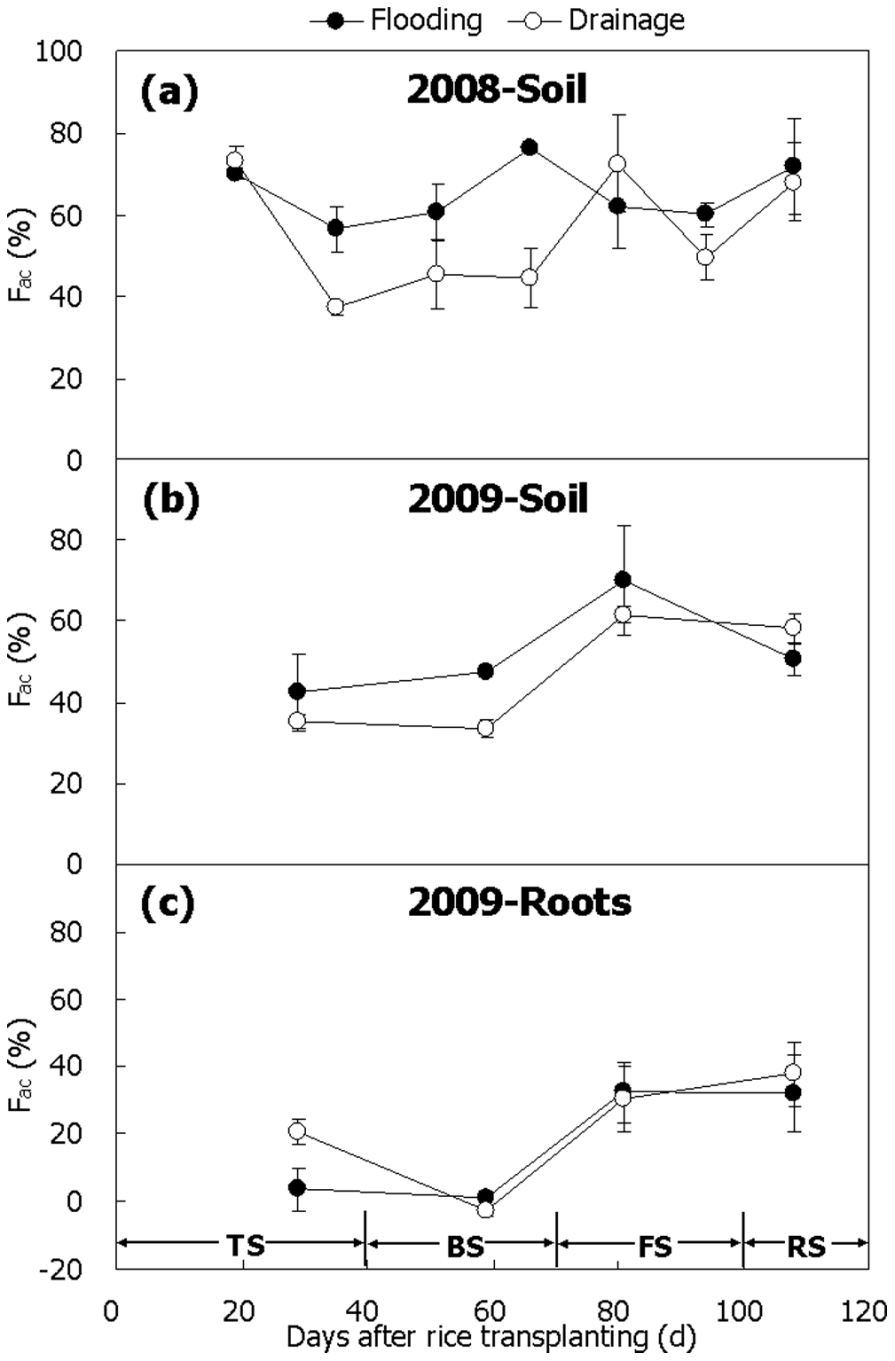
Relative contribution of acetate to total CH_4_ (*F_ac_*) in paddy soil and on rice roots. (a) 2008, (b and c) 2009. *F_ac_* was calculated with Eq. (7) using 1.079 for *α_CO2/CH4_* and –43‰ for δ^13^CH_4 (acetate)_. TS, BS, FS and RS represent tillering, booting, grain-filling and ripening stages, respectively. Bars represent standard errors (n = 3).

The processes of CH_4_ emission involved in CH_4_ production, oxidation and transportation in the fields were well identified by measuring stable carbon isotopes (Fig. 7). In paddy fields, besides the applied organic fertilizers, plant photosynthesis and degradation of soil organic carbon are the two most important substrate sources for methanogenesis [31]. As substrates for CH_4_ production, δ^13^C-value of organic carbon in the plant samples (–29‰) seemed to be slightly negative than that in the soil samples (–27‰) (Fig. 7). Previous observations also showed that organic carbon was slightly lighter in the plant than in the soil [10], [27], [32]. Intensive carbon isotope fractionation generally happens when methanogenic precursors form CH_4_. Around 10–20‰ occurs during CH_4_ production through acetate fermentation while 50–70‰ during CH_4_ production through H_2_/CO_2_ reduction [16], [33]. As a consequence, CH_4_ from the former (–60 ∼ –50‰) is usually more positive than that from the latter (as negative as –110‰) [34]. The CH_4_ produced in the soil was more ^13^C-enriched in Treatment Flooding than in Treatment Drainage (Fig. 3b, e) and also more (–65‰) than that on the roots (–80‰) in both treatments (Fig. 7). This shows that flooding, compared with drainage in the winter fallow season, increased the relative contribution of acetate to CH_4_ production and that aceticlastic methanogenesis in the soil was more important than that on the roots (Fig. 8). Early anaerobic measurements indicated that CH_4_ from the roots was more depleted in ^13^C than that from the soil [17], [27].

In rice-based ecosystems, the produced CH_4_, except for the portions oxidized and emitted into the atmosphere, is temporarily retained in the soil as entrapped CH_4_ and dissolved CH_4_ in soil pore water [35]. As mainly in the form of bubbles, CH_4_ in soil pore water probably remains unoxidized and is usually considered to be the original CH_4_ produced in the field in many reports [11], [12], [36]. However, Krüger et al. [10] found that CH_4_ in soil pore water poorly represented the produced CH_4_. In the present study, it might be partially oxidized as well in the rhizosphere during the periods of D30–60 in the 2009 season (Fig. 6d), though its mean δ^13^C-value lingered around –60‰ and close to that of the CH_4_ produced in anaerobic soil over the season (Fig. 7). When porewater CH_4_ is released through the soil-water interface in paddy fields, it will be considerably oxidized, leaving the remainder temporarily in the floodwater. Since ^12^CH_4_ is consumed faster than ^13^CH_4_ by soil microbes, the residual CH_4_ is then ^13^C-enriched [34]. As a consequence, floodwater CH_4_ (–45‰) was more ^13^C-enriched than porewater CH_4_ (–60‰). The observation of δ^13^C-value of the CH_4_ produced in aerobic soil being more positive than that in anaerobic soil (Fig. 7) further demonstrates that CH_4_ oxidation is intensive at the soil-water interface. In addition, rice roots can excrete O_2_ thus forming an important CH_4_-oxidizing zone in the rhizosphere. What is more, fresh rice roots per se have a strong CH_4_ oxidation capacity [10], [27], [37]. CH_4_ aerobically produced on the roots appeared to be more enriched in ^13^C (–45‰) than that in the soil (–55‰). It suggests that rice roots in the rhizosphere may be more important than the soil per se as driving force for CH_4_ oxidation, thus causing more ^12^CH_4_ consumed and leaving more ^13^CH_4_ remained (Fig. 7).

Aerenchymatic CH_4_ (∼ –47‰) was similar to oxidized CH_4_ in δ^13^C-value (Fig. 7), which demonstrates that it has been strongly oxidized in the rhizosphere. In Italian paddy fields, Krüger et al. [10] also found that it stayed around –50‰ throughout the rice-growing season. After being emitted through transportation of the plants, aerenchymatic CH_4_ was much heavier than emitted CH_4_ (Fig. 7), due to the fact that ^12^CH_4_ was transported from the plants at a faster rate than ^13^CH_4_ [38]. By subtracting δ^13^C-value of aerenchymatic CH_4_ from δ^13^C-value of emitted CH_4_ the transport fractionation by the plants is quantified [10]–[12]. In theory, the transport fractionation is relatively small due to small CH_4_ transport capacity of the plants at the beginning of the season. It gets strengthened together with the growth of the plants during the middle of the season but weakens again till the end of the season because of aging of the roots and plants. Consequently, value of fractionation (*ε_transport_*) was the lowest during the middle of the season, and relatively high at the beginning and the end of the season because it was shown as negative (Table 2). Moreover, it was averaged around –13‰, suggesting that water management in the winter fallow season has little effect on CH_4_ transport fractionation during the following rice-growing season. Similar ε_transport_ was also observed in other field experiments [10]–[12].

The δ^13^C-value of emitted CH_4_ fluctuated largely during the 2008 and 2009 rice seasons (Fig. 1b, f), and they were negatively related to CH_4_ emission in seasonal variation (r = –0.695 ∼ –0.546, *P*<0.01). An analogous relationship between them was also observed in other experiments [4], [26], which was considered to be the combined effect of CH_4_ production, oxidation and transport in the fields [26], [39], [40]. Although water management in the winter fallow season played a key role in CH_4_ emission from the rice fields (Fig. 1a, e), it had little impact on δ^13^C-value of emitted CH_4_ (Fig. 1b, f). For the two seasons, mean value was ∼ –60‰, being in the range of the measurements in previous report [26]. Compared with flooding, drainage had CH_4_ relatively more depleted in ^13^C (Fig. 3b, e), but the CH_4_ would become enriched in ^13^C again after it was oxidized because F_ox_-value in the latter was 5–15% higher (for detailed description, please see Section Effects on CH_4_ oxidation below). In addition, there was no obvious difference in *ε_transport_* between the two treatments (Table 2). Therefore, the ^13^C-depleted CH_4_ in Treatment Drainage was supposed to be offset by the higher fraction of CH_4_ oxidation, thus making the δ^13^C-value of emitted CH_4_ from the two treatments similar.

### Effects on CH_4_ Production

Previous studies demonstrated that water management in the winter fallow season significantly affected CH_4_ production during the following rice-growing season [8], [9]. In the present study, it showed an important effect on CH_4_ production of the fields by significantly affecting soil Eh. Methanogens are a kind of extreme anaerobic bacteria, which produce CH_4_ under strict reductive conditions. Compared to the fields flooded in the winter fallow season, the fields drained were probably much lower in population and activity of methanogens [41]–[43] and it generally took a longer time for methanogens to revive during the following rice-growing season [44]. Therefore, drainage delayed and decreased CH_4_ production in soil by significantly increasing soil Eh (Figs. 1c, g and 3a, d, Table 1). On roots as well, the lower the soil Eh, the higher the CH_4_ production (Table 1) because roots get to age and decay faster if they are constantly under a stronger reductive condition [45], [46]. A significant negative correlation between mean CH_4_ production and soil Eh (Table 1, r = –0.805, *P*<0.01 for soil and r = –0.994, *P*<0.01 for roots, respectively) better demonstrated that soil Eh significantly affected by water management in the winter fallow season was a key factor that influenced CH_4_ production. CH_4_ concentration in soil pore water being generally lower in Treatment Drainage than in Treatment Flooding (Table 1) further showed that drainage decreased CH_4_ production in the fields.

In paddy fields, CH_4_ mainly comes from acetotrophic and hydrogenotrophic methanogenesis. Methanol-dependent methanogenesis may possibly be another contributor to the total CH_4_ production, though, insignificant [47]. Relative contribution of acetotrophic methanogenesis (*F_ac_*) to the total CH_4_ production can be calculated by following Eq. (7) if a fractionation factor of *α_CO2/CH4_* = 1.079 is used for CO_2_-dependent methanogenesis and δ^13^CH_4 (acetate)_ = –43‰ is for acetate-dependent methanogenesis based on the following reports [17], [19]. In an Italian paddy soil, Fey et al. [19] found that *α_CO2/CH4_*, decreasing with increasing temperature, was 1.083 at 10°C, 1.079 at 25°C, and 1.073 at 50°C, which was in good agreement with the relationships in marine sediment [48] and methanogenic cultures [49]. Therefore, *α_CO2/CH4_* = 1.079 was applied because the temperature during the two seasons varied in the range of 20–30°C with an average of 24°C. On the other hand, Fey et al. [19] demonstrated that δ^13^CH_4 (acetate)_ increased with increasing temperature, e.g., from –50 ∼ –46‰ at 10°C to –45 ∼ –36‰ at 25°C, and to –43 ∼ –31‰ at 37°C. Moreover, the δ^13^CH_4 (acetate)_-values of –43 ∼ –36‰ have even been applied considerably to experiments in the fields during the rice-growing season [10]–[12], [36]. Due to lack of measurements, a constant value of –43‰ was hence used in the present study for better comparison with these reports. What is more important, it was more reasonable and suitable because δ^13^C-value of the soil organic carbon–substrate for methanogenesis, in this study was similar to those observed before [10], [17], [19]. Although they might be different in microbe habitats and varied with temperature and rice growth [10], [17], [19], the same values of *α_CO2/CH4_* and δ^13^CH_4 (acetate)_ above mentioned have also been used [4], [26], [27].

The findings show that variation of *F_ac_*-value in paddy soil during the 2008 rice season was similar to that during the 2009 rice season in pattern. That is, acetate-dependent methanogenesis dominated in the late season, while it was not so much important in the mid season, with *F_ac_*-value being over 60–70% and less than 40%, respectively (Fig. 8a, b). In Italian paddy fields, measurements also show that it was dominant at the end of the season [10]. Water management in the winter fallow season had an important impact on methanogenic pathways of paddy soil during the following rice-growing season. Generally, CH_4_ from acetate cleavage dominated in Treatment Flooding during the two rice seasons, having a mean *F_ac_*-value of 53–65%, which was 5–10% higher than in Treatment Drainage (Fig. 8a, b). Drainage increased production of oxidants, such as Fe^3+^ or sulphate, along with the increase in soil Eh [20], [50]. As a result, the growth and activity of methanogens was probably out-competed by iron- or sulphate-reducing bacteria [51], [52]. More importantly, acetotrophic methanogens seemed to be inhibited to a larger extent than hydrogenotrophic methanogens [20], [53]. This suggests that soil Eh is an important indicator of pathways of methanogenesis in paddy fields–the higher the soil Eh, the more inhibited the acetotrophic methanogens, and the less the contribution of acetate to the total methanogenesis. In the present study therefore, drainage in the winter fallow season significantly increased soil Eh (Fig. 1c, g) and obviously decreased methanogenesis in paddy soil compared to flooding (Fig. 3a, d), and hence the contribution of acetate-dependent methanogenesis, probably ascribed to the fact that acetate-utilizing methanogens was more inhibited by any increase in soil Eh (Fig. 1c, g) [20]. Intermittent irrigation during the rice-growing season significantly reduced the contribution of acetate to CH_4_ production, of which the finding could further verify this point of view [4].

The relative contribution of acetate-dependent methanogenesis on rice roots was similar to that in paddy soil in 2009, which was the lowest (almost 0%) in the mid season but rose up to the highest (∼40%) at the end of the season (Fig. 8c). In total, *F_ac_*-value was 1–32% in Treatment Flooding and 0–38% in Treatment Drainage, being much lower than that in the soil (Fig. 8). It indicates that methanogenesis on fresh rice roots is mostly from H_2_/CO_2_, and it is little affected by water management in the winter fallow season. Previous reports also show that *F_ac_*-value of rice roots was less than 40% in most of the season [10]. In an incubation experiment with rice roots D75–80 old, Conrad et al. [17] found that CH_4_ mainly came from H_2_/CO_2_-dependent methanogenesis as well throughout the entire observation, with an average *F_ac_*-value of 47%. Compared with that of soil, the relative contribution of acetate to the total methanogenesis on the roots was lower by approximately 30% (Fig. 8), which is likely attributed to the difference in population of their dominant methanogens [54]–[56]. More exact measurements using stable isotope probing techniques have further demonstrated that CH_4_ production on roots depends mainly on H_2_/CO_2_ reduction triggered by RC-I methanogens (Rice Cluster I Archaea) [57], [58]. On the other hand, organic carbon slightly lighter in plant samples than in soil samples might be a possible reason for *F_ac_*-value being much lower in paddy soil than on rice roots.

### Effects on CH_4_ Oxidation

CH_4_ oxidation in soil seemed to be highly influenced by soil temperature rather than water management in the winter fallow season. Firstly, no significant difference was observed between flooding and drainage in mean oxidation potential during the 2008 and 2009 seasons (Table 1). Secondly, it varied with soil temperature (Figs. 1d, h and 6a, b), and a positive relationship was observed over the two seasons (r = 0.703–0.859, *P*<0.05), which is in good agreement with the previous report [9]. An appropriate soil temperature favors growth of methane-oxidizing bacteria, thus enhancing their capacity of CH_4_ oxidation [59]. The higher the soil temperature within the range of 12.5–34.8°C, the higher the CH_4_ oxidation rate [60], which is consistent with our observations.

Considerable measurements on fresh roots have shown that the roots per se have a high CH_4_ oxidation capacity [10], [37], [61]. In the present study, CH_4_ oxidation on the roots was the strongest at the beginning of the season and weakened later on (Fig. 6c), being in agreement with the previous reports [10], [27]. Drainage compared to flooding in the winter fallow season significantly decreased CH_4_ oxidation potential on the roots (Fig. 6c), probably attributed to the effect of flooding highly increasing CH_4_ production (Fig. 4a). Higher concentration of CH_4_ stimulated growth and activity of the methanotrophs on the surface of the roots, thus raising their CH_4_ oxidation capacity [59].

**Figure 9:**
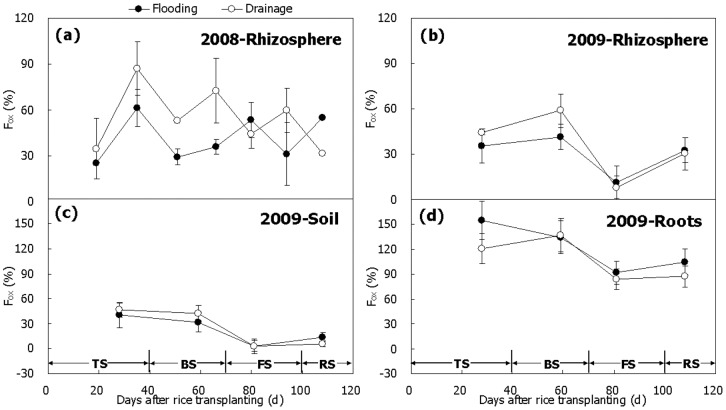
Temporal variation of the fraction of CH_4_ oxidized (*F_ox_*) in the rhizosphere and at the surfaces of paddy soil and rice roots. *F_ox_* in (a) and (b) was calculated with Eq. (8) using 1.038 for *α_ox_*, δ^13^C-values of CH_4_ anaerobically produced in soil (Fig. 3b, e) for δ^13^CH_4__(initial)_, and δ^13^C-values of emitted CH_4_ (Fig. 1b) minus –13.0‰ for both treatments in 2008 but (Fig. 1f) minus –13.3‰ for flooding and –12.8‰ for drainage in 2009 for δ^13^CH_4__(final)_. *F_ox_* in (c) and (d) was calculated in 2009 with Eq. (8) using 1.038 for *α_ox_*, δ^13^C-values of CH_4_ anaerobically produced in soil (Fig. 3e) and on roots (Fig. 4b) for δ^13^CH_4__(initial)_, and δ^13^C-values of CH_4_ aerobically produced in soil (Fig. 5b) and on roots (Fig. 5d) for δ^13^CH_4__(final)_. TS, BS, FS and RS represent tillering, booting, grain-filling and ripening stages, respectively. Bars represent standard errors (n = 3).

The fraction of CH_4_ oxidation (*F_ox_*) can be quantified by measuring δ^13^C-value of CH_4_ from various compartments of the paddy fields with a special model in case some parameters (*α_ox_* and *ε_transport_*) are already available [10]–[13]. The potential shift in the carbon isotopes during the CH_4_ oxidation (fractionation factor *α_ox_* = 1.025–1.038) was firstly determined in methanotrophs enriched cultures [62] and then considerably in landfill cover soils at a temperature of about 25°C [63]–[65]. Interestingly, the value of 1.025–1.038 has been widely applied to field conditions [10]–[12], [24], [25] though the knowledge of *α_ox_* in paddy soil is still incomplete. Very recently, we have found *α_ox_* = 1.025–1.033 at 28.3°C in a Chinese paddy soil [27]. Consequently, the value of 1.038 was used in the present study due to the similar temperature during the seasons, and more reasonable results would be obtained (Fig. 9). On the other hand, the transport fractionation factor *ε_transport_* was equivalent to the difference in ^13^C between emitted and aerenchymatic CH_4_ (Table 2), ranging from –16 to –11‰ in the 2009 season. In 2008 however, no corresponding measurements were performed. Nevertheless, the averaged value of –13‰ in 2009 was applied to the 2008 field data (Fig. 9), because it was also very close to previous observations [10]–[12]. Since fractionation factors (*α_ox_* and *ε_transport_*) are influenced by temperature, microbes, soil property, and rice growth [10], [64], more attention thereby need further be paid to getting reliable and exact values of CH_4_ oxidation in paddy fields.

Similar to the potentials of CH_4_ oxidation, the fraction of CH_4_ oxidized in the rhizosphere was relatively high (as high as 60–90%) in the first half of the rice growth period during the 2008 and 2009 seasons and relatively low (∼10–30%) in the remainder periods (Fig. 9a, b). In Italian paddy fields, measurements also show that CH_4_ oxidation was very important at the beginning of the season but became slight later, with *F_ox_*-value decreasing rapidly from approximately 40 to 0% [10], [20], [24]. Under unfertilized microcosms, Conrad and Klose [25] obtained that *F_ox_*-value decreased from about 15% in the beginning to about 5% at the end, which was probably attributed to nitrogen-limitation of the methanotrophs [10], [20], [24]. On the whole, mean value of *F_ox_* was 35–55% in Treatment Drainage, being 5–15% higher than that in Treatment Flooding during the 2008 and 2009 seasons (Fig. 9a, b). It suggests that compared to flooding in the winter fallow season drainage can increase the proportion of CH_4_ oxidized during the following rice-growing season. Probable reason was that drainage significantly decreased CH_4_ production (Fig. 3a, d) while it did not simultaneously affect CH_4_ oxidation in the field (Fig. 6a, b).

When CH_4_ in soil pore water passed through the soil-water interface into the floodwater, intensive signals of CH_4_ oxidation were observed by following changes in isotopic signature between them (Fig. 7). An obvious oxidation signal was also observed of the dissolved CH_4_ approaching to soil surface [11], [12]. Therefore, *F_ox_*-value was reasonably calculated based on δ^13^C-value of CH_4_ in pore water for δ^13^CH_4__(initial)_ and on δ^13^C-value of CH_4_ in floodwater for δ^13^CH_4__(final)_. It was high on D16 and D88, but relatively low on D47 and D50, especially in Treatment Flooding (Table 3). As the emission of CH_4_ in the fields goes absolutely through aerenchyma of the plants in the middle of the season, a very high percent of the CH_4_ is therefore consumed in the rhizosphere and a low percent oxidized at the soil-water interface (Table 3). On the contrary, CH_4_ emits into the atmosphere mainly through bubble ebullition and molecular diffusion in the early and the late rice-growing season, the CH_4_ is probably oxidized at the soil-water interface, showing a relatively high *F_ox_*-value as a consequence (Table 3). On the whole, there was no difference in mean *F_ox_*-value between the two treatments (47%) throughout the entire observational period, indicating that water management in the winter fallow season has little impact on CH_4_ oxidation at the soil-water interface. Notably, Krüger et al. [10] had even pointed out that porewater CH_4_ was a poor indicator of produced CH_4_. Therefore, CH_4_ in soil pore water on D47 and D50 in the present study might be oxidized partially as well. As a result, its δ^13^C-value was possibly not fit to stand for δ^13^CH_4__(initial)_, which would bias the estimation of CH_4_ oxidation therein. Actually, the CH_4_ produced in paddy fields would be mostly oxidized in the rhizosphere because over 90% of the CH_4_ is considered to emit into the atmosphere through the aerenchyma of the plants while less than 0.1% released via ebullition and diffusion [22], [23], [66]. Moreover, the absolute rates of CH_4_ oxidation at the soil-water interface were significantly lower than those in the rhizosphere [66]–[68]. Therefore, although the fraction of CH_4_ oxidized at the soil-water interface appears to be very high (Table 3), the amount of the CH_4_ must be significantly lower than that oxidized in the rhizosphere, and may be negligible.

**Table 3:** Fraction of CH_4_ oxidized (*F_ox_*) at the soil-water interface during the 2009 rice season (mean ± SD, n = 3).

Days after rice transplanting (d)	δ^13^CH_4__(initial)_ (‰)^a^		δ^13^CH_4__(final)_ (‰)^b^		*F_ox_* (%)^c^	
	Flooding	Drainage	Flooding	Drainage	Flooding	Drainage
16	–65.98±1.58	–59.47±3.86	–50.24±1.66	–49.62±3.09	45±9	28±11
47	–54.43±0.21	–54.53±0.31	–43.13±1.27	–37.59±0.00	32±3	48±1
50	–56.52±0.72	–58.71±0.75	–43.09±1.25	–41.12±0.06	38±4	50±2
88	–65.79±3.37	–63.69±0.01	–40.34±5.15	–42.08±2.60	72±0	62±5

a δ^13^C-values of CH_4_ in soil pore water (Fig. 2d);

b δ^13^C-values of CH_4_ in floodwater (Fig. 2d);

c Calculated with Eq. (8) using *α_ox_* = 1.038.

In lab conditions, the difference between anaerobic and aerobic CH_4_ productions in the soil was apparently attributed to CH_4_ oxidation at the soil-water interface [69]. In the present study, CH_4_ from aerobic incubation has undergone intensive oxidization, relative to CH_4_ produced anaerobically (Figs. 3d, e and 4a, b). This was reflected both in significantly low production rate (Fig. 5a, c) and more positive δ^13^C-value of the produced CH_4_ (Fig. 7). Therefore, the fraction of CH_4_ that was oxidized in aerobic condition could be estimated directly by using Eq. (8) based on δ^13^C-values of anaerobically produced CH_4_ for δ^13^CH_4__(initial)_ (Figs. 3e and 4b) and on δ^13^C-values of aerobically produced CH_4_ for δ^13^CH_4__(final)_ (Fig. 5b, d). Although little is known about the isotope fractionation when CH_4_ oxidation occurs on rice roots, *α_ox_* = 1.038 was tentatively used to estimate *F_ox_*-value thereupon as well as in paddy soil. Results show that a variation pattern of *F_ox_*-value was similar to that in the rhizosphere, which was the highest in the first half of the season but tended to get lower in the second (Fig. 9). For soil, *F_ox_*-value ranged from ∼5 to 50% and was slightly affected by water management in the winter fallow season (Fig. 9c). For roots however, it was over 100% in the most of the season, suggesting that fresh rice roots consume almost all the produced CH_4_ by themselves in lab conditions. Moreover, it was 15% lower in Treatment Drainage than in Treatment Flooding (Fig. 9d). It is a matter of fact that little CH_4_ was produced in aerobic incubation and it even became negative in growth at the end of the season (Fig. 3c). In addition, drainage significantly decreased the CH_4_ oxidation capacity of the field relative to flooding (Fig. 6c). Methanotrophs are found to attach closely to, or even live inside, rice roots [37], [70], [71]. The roots per se have a strong CH_4_ oxidation capacity indeed (Fig. 6c). On the other hand, it further indicates that value of *α_ox_* = 1.038 may be unreasonable for roots in estimation of *F_ox_*, because in field conditions, CH_4_ production directly or indirectly from the roots must not be completely oxidized and *F_ox_*-value should be lower than 100%. Therefore, more investigation of fractionation factor *α_ox_* in paddy soil, in particular on rice roots, should be performed to better quantify CH_4_ oxidation in the fields.

## Conclusions

Through the field and laboratory experiments, we investigated δ^13^C in every process of CH_4_ emission from rice fields as affected by water management in the winter fallow season and further estimated pathways of CH_4_ production and fraction of CH_4_ oxidation using the stable carbon isotope technique. Compared with flooding, drainage generally caused the produced CH_4_ depleted in ^13^C. Although drainage significantly decreased CH_4_ emission, it had little effect on δ^13^C-value of emitted CH_4_, as well as the transport fractionation factor *ε_transport_*. Acetate-dependent methanogenesis dominated in the soil in the late season, but H_2_/CO_2_-dependent methanogenesis occurred mostly on the rice roots over the season. Drainage decreased the contribution of acetate to CH_4_ production by 5–10%. In field conditions, ∼10–90% of the CH_4_ was oxidized in the rhizosphere, while ∼30–70% at the soil-water interface. In lab conditions, less a half of the CH_4_ was oxidized in the soil, while almost all on the roots. Moreover, CH_4_ oxidation was more important in the first half of the season as well as in the rhizosphere. Drainage increased the fraction of CH_4_ oxidized in the rhizosphere by 5–15%, which is possibly attributed to the fact that CH_4_ production decreased significantly while CH_4_ oxidation did not simultaneously. Measuring δ^13^C-values of the CH_4_ from different pools in the rice fields is useful for quantifying the methanogenic pathway and the fraction of CH_4_ oxidized in these fields. More importantly, it is useful for better understanding the processes of CH_4_ emission, which may provide useful information for setting up an isotope model. Such a model may be of a great help to national or global CH_4_ budget. Therefore, more attentions should be paid to the paddy fields with more different patterns of agricultural management at a larger scale.
